# The clinical significance of 10-m walk test standardizations in Parkinson’s disease

**DOI:** 10.1007/s00415-018-8921-9

**Published:** 2018-06-06

**Authors:** Beata Lindholm, Maria H. Nilsson, Oskar Hansson, Peter Hagell

**Affiliations:** 1 Department of Neurology and Rehabilitation Medicine, Skåne University Hospital, 205 02 Malmö, Sweden; 2 Clinical Memory Research Unit, Department of Clinical Sciences, Lund University, Malmö, Sweden; 3 Department of Health Sciences, Lund University, Lund, Sweden; 4 Memory Clinic, Skåne University Hospital, Malmö, Sweden; 5 The PRO-CARE Group, School of Health and Society, Kristianstad University, Kristianstad, Sweden

**Keywords:** Parkinson disease, The 10-m walk test, Test standardization, Falls, Prediction

## Abstract

**Background:**

The 10-m walk test (10MWT) is a widely used measure of gait speed in Parkinson’s disease (PD). However, it is unclear if different standardizations of its conduct impact test results.

**Aim of the study:**

We examined the clinical significance of two aspects of the standardization of the 10MWT in mild PD: static vs. dynamic start, and a single vs. repeated trials. Implications for fall prediction were also explored.

**Methods:**

151 people with PD (mean age and PD duration, 68 and 4 years, respectively) completed the 10MWT in comfortable gait speed with static and dynamic start (two trials each), and gait speed (m/s) was recorded. Participants then registered all prospective falls for 6 months.

**Results:**

Absolute mean differences between outcomes from the various test conditions ranged between 0.016 and 0.040 m/s (effect sizes, 0.06–0.14) with high levels of agreement (intra-class correlation coefficients, 0.932–0.987) and small standard errors of measurement (0.032–0.076 m/s). Receiver operating characteristic curves showed similar discriminate abilities for prediction of future falls across conditions (areas under curves, 0.70–0.73). Cut-off points were estimated at 1.1–1.2 m/s.

**Conclusions:**

Different 10MWT standardizations yield very similar results, suggesting that there is no practical need for an acceleration distance or repeated trials when conducting this test in mild PD.

## Introduction

The 10-m walk test (10MWT) is widely used and recommended as a measure of gait speed in Parkinson’s disease (PD). Its measurement properties are considered good and the test can be used to identify changes in gait speed in response to therapeutic interventions [1]. Furthermore, comfortable gait speed < 1.1 m per second (m/s) has been suggested as an important predictor of future falls in PD [2–5]. However, there are different standardizations for the conduct of the 10MWT, for example, measuring over different distances (10 or 6 m) and the inclusion or exclusion of an acceleration distance, i.e., dynamic vs. static start [1–3, 6].

According to general principles of measurement uncertainty, the best estimate of any measured quantity is the mean of repeated measures obtained under identical conditions [7]. Therefore, it is common to perform multiple trials and use the mean of these as the test result [8, 9]. For example, the 3-step falls prediction model (3-step model) prescribes the use of the mean value of two trials [2, 3]. However, it is unclear to what extent different 10MWT standardizations impact test outcomes and interpretations. In this respect, the clinical significance of any differences in outcome is not primarily related to the statistical significance resulting from null hypothesis testing and similar procedures (where, e.g., sample size is a major determinant). Instead, aspects such as effect sizes, absolute differences relative to estimated errors of measurement, and decision-making implications are more relevant to consider [10]. Therefore, we examined the clinical significance of two aspects of the standardization of conducting the 10MWT in mild PD: (1) using static vs. dynamic start and (2) using data from a single vs. two repeated trials. In addition, the implications of these standardizations in terms of prediction of future falls were explored.

## Methods

Participants were enrolled in a cohort study designed to study factors associated with falls and near falls in PD [11]. All people diagnosed with PD that received care at a south Swedish university hospital neurology outpatient clinic during 2007–2013 were considered eligible for inclusion (*n* = 359). Exclusion criteria were age above 80 years (*n* = 121), inability to understand instructions (*n* = 14), inability to stand without support (*n* = 22) and severe comorbidity (*n* = 11). Of the remaining 191 potential participants, 40 declined participation, leaving 151 participants (68 women) in the final study sample (Table 1). The Regional Ethical Review Board approved the study (Dnr 2011/768). All participants gave written informed consent.

**Table 1:** Sample characteristics (*n* = 151)

Age (years), mean (SD; min–max)	68 (9.6; 35–80)
Female gender, *n* (%)	68 (45)
PD duration (years), mean (SD; min–max)	4 (4; 0.1–17)
Stage of disease (HY), median (q1–q3; min–max)^a^	II (II–III; I–IV)
HY I, *n* (%)	12 (8)
HY II, *n* (%)	87 (57.5)
HY III, *n* (%)	45 (30)
HY IV, *n* (%)	7 (4.5)
Motor symptoms (UPDRS III), median (q1–q3; min–max) ^b^	12 (8–18; 1–46)
Cognition (MMSE), median (q1–q3; min–max) ^c^	28 (26–29; 18–30)
History of FOG, *n* (%)^d, *n* = 150^	63 (42)
Walking aids, *n* (%)	19 (13)
Cane	3 (2)
Crutch	3 (2)
Walker	13 (9)
Individuals with one or more prospective falls, *n* (%)^e, *n*=146^	47 (32)

*PD* Parkinson’s disease, *q1–q3* 1st–3rd quartile, *HY* Hoehn and Yahr stage of PD, *UPDRS III* part III (motor examination) of the Unified Parkinson’s Disease Rating Scale, *MMSE* mini-mental state examination, *FOG* freezing of gait

^a^Stages range between I (mild unilateral disease) and V (confined to bed or wheelchair unless aided)

^b^Scores range 0–108 (0 = better)

^c^Scores range 0–30 (30 = better)

^d^People scoring ≥ 1 on the self-administered Freezing of Gait Questionnaire (FOGQsa), item 3 (*Do you feel that your feet get glued to the floor while walking, making a turn or when trying to initiate walking (freezing)?*) were categorized as having FOG

^e^As determined using a prospective falls diary during a 6-month follow-up (see Lindholm et al. [11] for details)

Detailed descriptions regarding the overall procedures are available elsewhere [11]. Participants were assessed during an outpatient visit, scheduled at a time of day when they reported to typically feel at best. The 10MWT was conducted in comfortable gait speed following a verbal start command. Timing according to static start (ss) was done over the first 10 m, and timing according to dynamic start (ds) was done between 2 and 12 m. Walking aids were permitted. Walking time was measured to the nearest 0.001 s (s) using a digital stopwatch (Origo, model 365,510) when the lead foot crossed the respective markers (at 0, 2, 10 and 12 m), and rounded to the closest 0.01 s. Two trials (t1 and t2) each of ss and ds were conducted. Gait speed was calculated as m/s.

In addition, participants were assessed regarding various aspects of their PD, including disease severity (Hoehn and Yahr staging (HY)) [12], motor symptoms (part III of the Unified PD Rating Scale (UPDRS)) [13], and cognition (the mini-mental state examination (MMSE)) [14]. Freezing of gait (FOG) was investigated with item 3 of the self-administered Freezing of Gait Questionnaire (FOGQsa) (*Do you feel that your feet get glued to the floor while walking, making a turn or when trying to initiate walking (freezing)?*). Those scoring ≥ 1 were categorized as having FOG [15, 16].

As the last step during the outpatient visit, participants were instructed to register all consecutive falls and near falls during the following 6 months [17]. They were provided with a diary folder consisting of pre-printed pages for recording the date and time of every event and questions clarifying whether the incident was a fall. The question was phrased as follows: *Did you fall in such a way that your body hit the ground?* Falls were defined as “an unexpected event in which the participants come to rest on the ground, floor, or lower level” [17]. The definition of a fall was thoroughly described during the outpatient visit. All participants were telephoned monthly to ensure that registrations had been completed according to instructions. During the last telephone call, they were requested to return the diary folder in a pre-stamped envelope.

### Analyses

Data were analysed using IBM SPSS version 22 (IBM Corp., Armonk, NY) with the alpha level of significance set at 0.05 ( two tailed). Paired sample *t* tests were used to explore differences between different standardizations of the 10MWT (ss vs. ds) conducted at t1 and t2, as well as the mean values of these (*M*_ss_ vs. *M*_*ds*_). Similarly, we examined the differences between trials (t1 vs. t2), and between t1 and mean t1 and t2 values (*M*_t1, t2_) for ss and ds, respectively. Effect sizes (ESs) were computed using Cohen’s *d*; ESs were interpreted as small (0.20), moderate (0.50), large (0.80) and very large (1.3) [10]. Intra-class correlation (ICC) coefficients (two-way mixed effects model, absolute agreement, single measure) were calculated to determine the agreement between test conditions, and the standard error of measurement (SEM) was estimated (SD_t1_ × √[1-ICC]). The analyses were performed for the full sample (*n* = 151). In addition, exploratory subgroup analyses were conducted among (1) people with a history of FOG (*n* = 63), (2) those in HY stage IV and/or using walking aids during testing (*n* = 21), (3) those who self-rated their motor status as “off” during testing (*n* = 8).

Receiver operating characteristic (ROC) curve analysis was used to determinate optimal gait speed cut-off points for prediction of one or more future falls in the full group (*n* = 151). The optimal point is that with the highest true-positive (sensitivity) and lowest false-positive (1-specificity) values. The areas under the ROC curves (AUROCs) can range between 0 and 1, where an AUROC <0.5 indicates that a test performs worse than chance; AUROCs ≥ 0.7 are acceptable, with values between 0.7 and 0.9 and >0.9 considered moderate and high, respectively [18, 19]. Values (m/s) associated with the highest Youden index (sensitivity + specificity − 1) were estimated as the optimal cut-off points to discriminate between those with and without future falls [19].

## Results

All 151 participants completed the 10MWT testing. Sample characteristics are summarized in Table 1. Participants’ mean (SD) age and PD duration were 68 (9.6) and 4 (4) years, respectively; their median (q1–q3) Hoehn and Yahr (HY) stages were II (II–III). Freezing of gait (FOG) was experienced by 63 (42%) participants, and 21 (14%) were in HY stage IV or used walking aids during testing. At the time of assessments, 143 participants (95%) rated their motor status as “on” or “on with dyskinesias” and 8 (5%) rated it as “off”. One hundred forty-six (97%) individuals completed prospective follow-up during the 6-month period. Forty-seven of those (32%) reported at least one fall and 28 (19%) reported more than one fall.

There were statistically significant differences (*P* < 0.001) in gait speed at all instances in the full group (*n* = 151). Absolute mean differences between outcomes from the various test conditions were generally small, ranging between 0.016 and 0.040 m/s (ESs 0.06–0.14) with high levels of agreement (ICC 0.932–0.987) and small measurement errors (SEM 0.032–0.076 m/s). Further details are provided in Table 2. Similar results were also obtained when repeating these analyses among subgroups of individuals with a history of FOG (*n* = 63; Table 3) and those in HY stage IV or using walking aids during testing (*n* = 21; Table 4), as well as among those who self-rated their motor status as “off” during testing (*n* = 8; data not shown).

**Table 2:** Gait speed characteristics according to different standardizations of the 10-m walk test in PD (*n* = 151)

	Mean (SD)^a,b^	Mean difference (95% CI)	ES^c^	ICC (95% CI)^d^	SEM ^a^
Static vs. dynamic start					
t1_ss_ vs. t1_ds_ (*n* = 150)	1.111 (0.292) vs. 1.094 (0.277)	0.017 (0.08, 0.0259)	0.06	0.980 (0.971, 0.987)	0.041
t2_ss_ vs. t2_ds_ (*n* = 149)	1.153 (0.298) vs. 1.129 (0.286)	0.024 (0.011, 0.357)	0.08	0.964 (0.946, 0.975)	0.057
*M*_ss_ vs. *M*_ds (_*n* = 149)	1.132 (0.290) vs. 1.112 (0.278)	0.020 (0.012, 0.028)	0.07	0.983 (0.970, 0.989)	0.037
Static start (*n* = 150)					
t1_ss_ vs. t2_ss_	1.114 (0.291) vs. 1.154 (0.297)	− 0.040 (− 0.057, − 0.024)	0.14	0.932 (0.890, 0.956)	0.076
t1_ss_ vs. *M*_t1,t2_	1.114 (0.291) vs. 1.134 (0.290)	− 0.020 (− 0.028, − 0.012)	0.07	0.982 (0.971, 0.989)	0.039
Dynamic start (*n* = 149)					
t1_ds_ vs. t2_ds_	1.096 (0.277) vs. 1.129 (0.286)	− 0.033 (− 0.047, − 0.020)	0.12	0.950 (0.917, 0.968)	0.062
t1_ds_ vs. *M*_t1,t2_	1.096 (0.277) vs. 1.112 (0.278)	− 0.016 (− 0.024, − 0.010)	0.06	0.987 (0.978, 0.992)	0.032

*PD* Parkinson’s disease, *m/s* meters per second, *SD* standard deviation, *CI* confidence interval, *ES* effect size, *ICC* intra-class correlation, *SEM* standard error of measurement (SD_t1_ × √1– ICC), *t1*_*ss*_ trial 1 with static start, *t1*_*ds*_ trial 1 with dynamic start, *t2*_*ss*_ trial 2 with static start, *t2*_*ds*_ trial 2 with dynamic start, *M*_*ss*_ mean value of trials 1 and 2 with static start, *M*_*ds*_ mean value of trials 1 and 2 with dynamic start, *M*_*t1,t2*_ mean value of trials 1 and 2

^a^Data are in m/s

^b^*P* < 0.001 in all instances (paired samples *t* tests)

^c^Cohen’s *d (*calculated on between-test differences*)*

^d^Two-way mixed effects model (absolute agreement, single measure)

**Table 3:** Gait speed characteristics according to different standardizations of the 10-m walk test in individuals with PD and history of FOG (*n* = 63)

	Mean (SD)^a,b^	Mean difference (95% CI)	ES^**c**^	ICC (95% CI)^d^	SEM ^a^
Static vs. dynamic start					
t1_ss_ vs. t1_ds (_*n* = 63)	0.943 (0.284) vs. 0.934 (0.272)	0.009 (− 0.008, 0.022)	0.03	0.977 (0.963, 0.986)	0.043
t2_ss_ vs. t2_ds_ (*n* = 62)	0.990 (0.308) vs. 0.971 (0.298)	0.019 (− 0.004, 0.044)	0.06	0.950 (0.918, 0.970)	0.068
*M*_ss_ vs. *M*_ds_ (*n* = 62)	0.968 (0.293) vs. 0.954 (0.282)	0.014 (− 0.002, 0.029)	0.05	0.977 (0.962, 0.986)	0.044
Static start (*n* = 62)					
t1_ss_ vs. t2_ss_	0.945 (0.287) vs. 0.990 (0.308)	− 0.045 (− 0.076, − 0.021)	0.15	0.934 (0.867, 0.964)	0.073
t1_ss_ vs. *M*_t1,t2_	0.945 (0.287) vs. 0.968 (0.293)	− 0.023 (− 0.036, − 0.011)	0.08	0.982 (0.963, 0.991)	0.039
Dynamic start (*n* = 62)					
t1_ds_ vs. t2_ds_	0.937 (0.274) vs. 0.971 (0.298)	− 0.034 (− 0.058, − 0.008)	0.12	0.937 (0.891, 0.963)	0.068
t1_ds_ vs. *M*_t1,t2_	0.937 (0.274) vs. 0.954 (0.282)	− 0.017 (− 0.029, − 0.004)	0.06	0.983 (0.970, 0.985)	0.036

*PD* Parkinson’s disease, *FOG* freezing of gait, *m/s* meters per second, *SD* standard deviation, *CI* confidence interval, *ES* effect size, *ICC* intra-class correlation, *SEM* standard error of measurement (SD_t1_ × √1– ICC), *t1*_*ss*_, trial 1 with static start, *t1*_*ds*_ trial 1 with dynamic start, *t2*_*ss*_ trial 2 with static start, *t2*_*ds*_, trial 2 with dynamic start, *M*_*ss*_ mean value of trials 1 and 2 with static start, *M*_*ds*_ mean value of trials 1 and 2 with dynamic start, *M*_*t1,t2*_ mean value of trials 1 and 2

^a^Data are in m/s

^b^*P* < 0.01 in all instances (paired samples *t* tests) in all instances but comparisons between static vs. dynamic start (*P* ≥ 0.082)

^c^Cohen’s *d (*calculated on between-test differences*)*

^d^Two-way mixed effects model (absolute agreement, single measure)

**Table 4:** Gait speed characteristics according to different standardizations of the 10-m walk test in individuals with PD in HY stage IV or using walking aids during testing (*n* = 21)

	Mean (SD)^a,b^	Mean difference (95% CI)	ES^c^	ICC (95% CI)^d^	SEM^a^
Static vs. dynamic start					
t1_ss_ vs. t1_ds_ (*n* = 21)	0.700 (0.189) vs. 0.698 (0.154)	0.002 (− 0.029, 0.036)	0.01	0.941 (0.853, 0.977)	0.046
t2_ss_ vs. t2_ds_ (*n* = 20)	0.722 (0.205) vs. 0.701 (0.160)	0.021 (− 0.015, 0.057)	0.11	0.920 (0.802, 0.969)	0.058
*M*_ss_ vs. *M*_ds_ (*n* = 20)	0.694 (0.154) vs. 0.706 (0.196)	− 0.012 (− 0.021, 0.045)	0.07	0.931(0.829, 0.974)	0.040
Static start (*n* = 20)					
t1_ss_ vs. t2_ss_	0.690 (0.189) vs. 0.722 (0.205)	− 0.032 (− 0.061, − 0.003)	0.16	0.947 (0.838, 0.981)	0.044
t1_ss_ vs. *M*_t1,t2_	0.690 (0.190) vs. 0.706 (0.196)	− 0.016 (− 0.030, − 0.002)	0.08	0.986 (0.954, 0.995)	0.022
Dynamic start (*n* = 20)					
t1_ds_ vs. t2_ds_	0.688 (0.152) vs. 0.701 (0.160)	− 0.013 (− 0.040, 0.013)	0.08	0.943 (0.857, 0.978)	0.036
t1_ds_ vs. *M*_t1,t2_	0.688 (0.152) vs. 0.695 (0.154)	− 0.007 (− 0.020, 0.006)	0.05	0.985 (0.962, 0.994)	0.019

*PD* Parkinson’s disease, *HY* Hoehn and Yahr stage of PD, *m/s* meters per second, *SD* standard deviation, *CI* confidence interval, *ES* effect size, *ICC* intra-class correlation, *SEM* standard error of measurement (SD_t1_ × √1– ICC), *t1*_*ss*_ trial 1 with static start, *t1*_*ds*_ trial 1 with dynamic start, *t2*_*ss*_ trial 2 with static start, *t2*_*ds*_ trial 2 with dynamic start, *M*_*ss*_ mean value of trials 1 and 2 with static start, *M*_*ds*_ mean value of trials 1 and 2 with dynamic start, *M*_*t1,t2*_ mean value of trials 1 and 2

^a^Data are in m/s

^b^*P* ≥ 0.298 in all instances (paired samples *t* tests) but t2_ss_ vs. t2_ds_ (static vs. dynamic start; *P* = 0.031), t1_ss_ vs. t2_ss_ and t1_ss_vs. *M*_t1,t2_ (static start; *P* = 0.031)

^c^Cohen’s *d* (calculated on between-test differences)

^d^Two-way mixed effects model (absolute agreement, single measure)

Forty-seven of 151 participants (32%) reported at least one prospective fall during the 6-month follow-up. ROC curve analyses showed similar discriminate abilities for future falls across the various 10MWT test conditions (AUROC, 0.70–0.73). The Youden index ranged between 0.37 and 0.39, with corresponding cut-off points estimated at 1.1–1.2 m/s (Table 5).

**Table 5:** Discriminant ability of the 10-m walk test (10MWT) for identification of individuals with prospective falls (*n* = 146)

	AUROC^b^ (95% CI)	Cut-off point (m/s)	Sensitivity^c^	Specificity^d^	Youden index^e^
Static start					
t1	0.72 (0.64, 0.81)	1.1	0.70	0.69	0.39
t2	0.72 (0.63, 0.81)	1.2	0.70	0.69	0.39
*M*_t1,t2_	0.73 (0.64, 0.81)	1.1	0.67	0.70	0.37
Dynamic start					
t1	0.71 (0.62, 0.80)	1.1	0.72	0.66	0.38
t2	0.70 (0.60, 0.79)	1.1	0.70	0.67	0.37
*M*_t1,t2_	0.70 (0.61, 0.80)	1.1	0.70	0.68	0.38

*m/s* meters per second, *t1* trial 1, *t2* trial 2, *M*_*t1,t2*_ mean value of t1 and t2

^a^As determined using a prospective fall diary during a 6-month follow-up (see Lindholm et al. 2015 for details)

^b^Area under the receiver operating characteristic curves of t1, t2 and *M*_t1,t2_ during 10MWT with static and dynamic start

^c^The proportion of people with prospective falls who had a positive result (scored above the cut-off point)

^d^The proportion of people without prospective falls who had a negative result (scored below the cut-off point)

^e^Sensitivity + specificity− 1

## Discussion

We examined the effects of different standardizations of the 10MWT in people with relatively mild PD. Although comparisons showed statistically significant differences between testing conditions, the sizes of these differences were small and the various test results showed high levels of agreement. The measurement error (SEM) has been suggested as a distribution-based minimal important difference (MID) indicator, at and above which differences in outcomes reflect differences of clinical interest [20]. In this study, the SEM values exceeded absolute mean differences by factors of 1.9–2.4. When considering the three subgroups, SEM values exceeded absolute mean differences by factors of up to 23.

It has been suggested that FOG may affect the outcome of the 10MWT [1]. Indeed, individuals with FOG walked slower when compared to the whole group. Although we did not observe any FOG during testing, 10MWT-based gait speed estimates were in agreement across standardizations also among people who reported having FOG. This may have been due to the use of a start command or straight walkway during 10MWT.

Taken together, these observations provide evidence that observed differences across test conditions can be considered clinically trivial. Previous anchor-based MID estimates in non-PD samples have ranged from 0.10 to 0.16 m/s [1]. Taking these estimates into account, the clinical meaningfulness of the observed differences diminishes even further.

In accordance with assumptions regarding the mean of repeated measures obtained under identical conditions as the best estimate of any measured quantity [7], mean values from trials 1 and 2 yielded smaller differences, effect sizes and SEM values together with larger ICCs than single-observation data from trials 1 and 2. However, given that the observed differences were very small, it is questionable if they are of any clinical significance. Therefore, unless there is a specific reason to maximise precision, our findings suggest that a single trial is sufficient for most practical situations. Similarly, the negligible differences between static and dynamic start suggest that the acceleration distance does not appear to affect the estimated gait speed considerably. The finding that repeated trials and dynamic start do not appear to have any practical impact on 10MWT-based outcomes simplifies its conduct and should facilitate its use in routine clinical practice.

The observations discussed above were also corroborated when exploring the implications of different 10MWT standardizations in terms of predicting future falls. The discriminant abilities of both static and dynamic start were very similar for both single trials and mean values. It is also noteworthy that trial 1 values and mean (trials 1 and 2) values of both static and dynamic start identified 1.1 m/s as an optimal cut-off point. This finding is in line with the suggested cut-off point for comfortable gait speed as a predictor in the 3-step model [2, 3]. However, while the 3-step model prescribes the use of the mean of two trials, our observations suggest that a single trial with static start will suffice.

Although this study strengthens the current evidence base regarding the conduct of the 10MWT in PD, it has some limitations that should be acknowledged. The study involved people with relatively mild PD, and people above the age of 80 years were not included. Our findings may, therefore, not be applicable to older people with mild PD and those in more advanced PD stages. Second, while factors such as HY stage IV, the use of walking aids or being “off” during testing did not appear to affect agreements between different standardizations of the 10MWT, these results are limited by relatively small numbers of individuals in the respective subgroups. Therefore, further investigations in larger samples will be needed for firmer conclusions. Furthermore, we investigated static vs. dynamic start with a start command and the use of a single vs. the mean of two repeated trials. However, other standardizations also exist, e.g., with timing over the mid 6 m walking distance and the mean m/s from three repeated trials. The clinical significance of these and other standardizations of the 10MWT need to be determined in additional studies.

With these considerations in mind, we conclude that there does not appear to be a need for using an acceleration distance or repeated trials in the clinical conduct of the 10MWT among people with mild PD. Further studies are warranted to explore the generalizability of these findings in different PD samples and across other standardizations of the 10MWT.
